# Prophylactic vs. Intermediate Tinzaparin Dosage for the Thromboprophylaxis of Acutely Ill Medical Patients at High Risk of Venous Thromboembolism

**DOI:** 10.3390/medsci13040291

**Published:** 2025-11-27

**Authors:** Karolina Akinosoglou, Stamatia Tsoupra, Ioannis Chandroulis, Eleni Polyzou, Vasiliki Dimakopoulou, Konstantinos Moulakakis, Angelos Perperis, Eleni Karlafti, Elvira Ztriva, Vasileios Patriarcheas, Periklis Davlouros, Georgia Kaiafa, Christos Savopoulos

**Affiliations:** 1 Department of Medicine, University of Patras, 26504 Rio, Greece; stamtsoupra@gmail.com (S.T.); polyzou.el@gmail.com (E.P.); dimakopoulou.vasilina@gmail.com (V.D.); pdav@upatras.gr (P.D.); 2 Department of Internal Medicine, University General Hospital of Patras, 26504 Rio, Greece; 3 Division of Infectious Diseases, University General Hospital of Patras, 26504 Rio, Greece; 4 School of Social Sciences, Hellenic Open University, 26331 Patra, Greece; std528050@ac.eap.gr; 5 Department of Vascular Surgery, University General Hospital “Attikon”, 12462 Chaidari, Greece; konmoulakakis@yahoo.gr; 6 Division of Cardiology, University General Hospital of Patras, 26504 Rio, Greece; angelosperperis@msn.com; 7 First Propaedeutic Department of Internal Medicine, AHEPA University General Hospital of Thessaloniki, Aristotle University of Thessaloniki, 54636 Thessaloniki, Greece; linakarlafti@hotmail.com (E.K.); elztriva@gmail.com (E.Z.); vpatriar@gmail.com (V.P.); gdkaiafa@yahoo.gr (G.K.); chrisavopoulos@gmail.com (C.S.); 8 Department of Medicine, Aristotle University of Thessaloniki, 54636 Thessaloniki, Greece

**Keywords:** venous thromboembolism, tinzaparin, thromboembolic events, prophylactic dosage, intensified prophylaxis, intermediate dosage

## Abstract

Background/Objectives: Venous thromboembolism (VTE) is the third most common cardiovascular condition, with higher rates among hospitalized patients. The limited efficacy of universal prophylaxis strategies has led to individual VTE risk assessments approaches. The main objective of this study was to assess outcomes in high-risk patients for VTE who receive prophylactic vs. intermediate, weight-adjusted doses of tinzaparin for thromboprophylaxis. Methods: This was a retrospective study assessing adult patients hospitalized with acute medical disease in a tertiary university hospital from January 2022–2024. Patients were included if found to be at high risk for VTE—as this reflected in Padua Prediction Score (PPS) ≥ 4—and received prophylactic versus intermediate dosage of tinzaparin. Data were collected from patients’ files and analyzed using appropriate statistical methods. Results: In total, 286 patients were included, of whom 160 received prophylactic and 126 intermediate tinzaparin dosage. The groups were comparable, except for arterial thrombosis history, central venous catheter presence, and median PPS. Patients receiving prophylactic doses exhibited significantly higher mortality rates (20.62 vs. 7.14, *p* = 0.002), increased length of stay (LOS) (6 vs. 4, *p* < 0.001), and prolonged treatment durations (5 vs. 3, *p* = 0.003) compared to patients receiving intermediate dosages. Univariate analysis revealed significant associations between mortality and tinzaparin dose (OR = 3.38, *p* = 0.002), age (OR = 1.03, *p* = 0.017), LOS (OR = 1.07, *p* = 0.001), PPS (OR = 1.62, *p* < 0.001), Charlson Comorbidity Index (CCI) (OR = 1.27, *p* < 0.001), and prior thrombotic events (OR = 2.27, *p* = 0.028). In multivariate analysis, tinzaparin dose (OR = 2.58, *p* = 0.035), age (OR = 1.04, *p* = 0.033), LOS (OR = 1.10, *p* < 0.001), and PPS (OR = 1.33, *p* = 0.038) remained independent predictors of mortality. Conclusions: These findings reveal that intermediate tinzaparin dosing is a more effective and safe approach in high-risk for VTE hospitalized patients, emphasizing the need for personalized VTE management.

## 1. Introduction

Venous thromboembolism (VTE) represents the third most frequent cardiovascular condition, with an annual occurrence rate of 1–2 in 1000 among middle-aged individuals, increasing significantly to 1% annually in the elderly population [1]. Around half of all community-based VTE cases are linked to recent or ongoing hospitalization, primarily due to surgery or acute medical conditions, including cancer and its treatment, trauma, prolonged immobility, use of central venous catheters, prior VTE episodes, advanced age, and obesity [2]. Nearly all hospitalized patients have at least one risk factor for VTE, with about 40% having three or more [3]. The elevated risk of VTE persists even after hospital discharge [4], underscoring the importance of awareness and proactive management of these risk factors to minimize the threat. Hospitalization due to acute illness presents a critical opportunity to implement preventative strategies.

Medical inpatients display diverse VTE risk profiles, but traditional prevention methods have often employed universal or group-based strategies [5,6]. However, recent research on medically ill hospitalized patients suggests that these broad approaches have minimal effect on reducing VTE incidence [7,8]. This limited impact may stem from several factors: (1) shorter hospital stays and less effective thromboprophylaxis regimens compared to older studies demonstrating significant benefits, or insufficient follow-up duration in newer research [9,10]; (2) inappropriate prophylaxis—where low-risk patients are overtreated and high-risk patients are undertreated—leading to an unfavorable balance between risks and benefits; or (3) suboptimal use of prophylaxis by clinicians due to concerns about bleeding risks or the perception that the patient’s VTE risk does not justify preventive measures [11].

In light of these insights, a shift in approach to VTE risk assessment and prevention has emerged. The new paradigm emphasizes personalized prophylaxis tailored to individual VTE and bleeding risk profiles, encouraging clinicians to move away from one-size-fits-all strategies. Over the past decade, several quantitative risk assessment models (RAMs) for VTE have been developed for medically ill patients [12]. One extensively studied model is the Padua Prediction Score (PPS) [13]. This model has been validated by independent studies and has demonstrated the ability to distinguish between medically ill patients and identify those at increased risk of VTE [12,14]. The rate of symptomatic VTE for low-risk patients (PPS < 4) was 0.3%, whereas the respective risk for high-risk patients (score ≥ 4) was 2.2 (receiving adequate in-hospital thrοmbοрrοрhуlaхis) and 11% (not receiving adequate in-hospital thrοmbοрrοрhуlахiѕ) [13,15].

Although international guidelines recommend the use of pharmacological VTE prophylaxis for patients with acute medical illness [16], the incidence of thromboembolic complications in these patients—despite prophylaxis—has led many hospitals to adopt flexible strategies [16]. These strategies often involve increasing the standard prophylactic doses of anticoagulants, including low-molecular-weight heparins (LMWHs), to intermediate doses, depending on the presence of risk factors [16]. Treating physicians must carefully evaluate the benefits and risks of anticoagulant therapy, considering both thrombosis and bleeding risks, in a personalized manner for each medically ill patient [16].

LMWHs exhibit distinct characteristics due to the various techniques employed in their production [17,18]. These differences in molecular structure and pharmacological behavior contribute to variations in their clinical effectiveness and safety profiles [17,18]. Consequently, each LMWH should be regarded as a unique compound. Notably, tinzaparin is the only LMWH manufactured through enzymatic hydrolysis using heparinase [19,20]. This specific production method imparts tinzaparin with unique features, such as higher anti-IIa activity, a distinct anti-Xa/Anti-IIa activity ratio, increased release of Tissue Factor Pathway Inhibitor (TFPI), reduced reliance on renal function for clearance, and more complete neutralization by its antidote when necessary [21]. These specialized properties, particularly in anti-IIa activity and the stimulation of TFPI release from endothelial cells, suggest that tinzaparin may have broader applications. Given the significant role of TFPI in various vascular, inflammatory, cardiovascular, hematological, and oncological conditions [22,23,24,25], tinzaparin’s potential utility could extend beyond its established anticoagulant effects.

The main objective of this study was to assess outcomes of VTE and bleeding in high-risk VTE patients who have acute pathological disease and receive prophylactic vs. intermediate weight-adjusted doses of tinzaparin for thromboprophylaxis. Objectives include the evaluation of which doses are administered during hospitalization and related incidence of thrombotic and bleeding events. In parallel, we aimed to determine the frequency of the different risk factors for thrombotic or bleeding episodes in hospitalized pathological patients to determine the frequency of the different risk factors included in the PPS model in hospitalized pathological patients with a Padua score ≥ 4 and the duration of their hospitalization.

## 2. Materials and Methods

A retrospective study was performed in the medical ward of a tertiary university hospital from January 2022–January 2024. Data was collected after reviewing the medical records of all patients who received thromboprophylaxis with prophylactic or intermediate weight-adjusted doses of tinzaparin during their hospitalization. As this was a retrospective study including all consecutive eligible admissions during the two-year-period, the cohort size was not predefined by sample-size calculation. Nevertheless, a post hoc power estimation confirmed sufficient power (>80%) for the primary endpoint (mortality difference between groups). The study was performed according to Good Clinical Research Practice and the Declaration of Helsinki and was approved by the local ethics committee and respective institutional review board (94/12.02.24, approval date 12 February 2024). The study was retrospective and observational; therefore, registration on ClinicalTrials.gov or similar platforms was not applicable.

### 2.1. Study Inclusion/Exclusion Criteria

Adult patients admitted to the hospital with acute pathological disease who have a PPS ≥ 4 (Table 1) [13] and who have been administered thromboprophylaxis with prophylactic or intermediate doses of tinzaparin during their hospitalization were included in this study. Although all patients had PPS ≥ 4, dosing decisions were individualized by attending physicians considering age, comorbidities, renal function, clinical trajectory, frailty and mobility status, and perceived bleeding risk. Consequently, prophylactic or intermediate tinzaparin was prescribed according to clinical judgment. Acute pathological disease was defined as disease with rapid symptom onset that required immediate or short-term medical intervention. Examples include, but are not limited to, the following: infections such as pneumonia, influenza, cardiovascular emergencies, e.g., myocardial infarction, hypertensive crisis, etc.; neurological conditions, e.g., stroke, seizures, etc.; acute respiratory issues, e.g., asthma exacerbations; gastrointestinal conditions, e.g., acute appendicitis, pancreatitis, etc.; metabolic disturbances, e.g., diabetic ketoacidosis, etc.; and acute kidney injury. Patients were excluded if admitted to the hospital with acute pathological disease, who had a PPS < 4, aged < 18, were pregnant, had an admission diagnosis of acute pulmonary embolism (PE) or deep vein thrombosis (DVT), severe renal impairment, PLT ≤ 100,000/mm^3^, or a disease with a life expectancy of less than 24 h from admission, regardless of treatment provision.

### 2.2. Data Collection

Data were collected from patients’ files, including demographic data (gender, date of birth, weight, height, etc.), medical history and pathological characteristics (thrombotic and bleeding history, risk factors, recent surgery, comorbidities, chronic medication use, etc.), disease characteristics, PPS anticoagulation data, thrombotic or bleeding episodes during hospitalization, other adverse events related to anticoagulation, and duration of hospitalization.

Intermediate weight-adjusted doses of tinzaparin were defined as doses of 50–75% of the full therapeutic dose, which is 175 anti-Xa IU/Kg, subcutaneously (SC), once daily (OD), e.g., approximately 90–130 Anti-Xa IU/Kg. More specifically, they are defined as follows: <50 kg: 4500 IU daily (subcutaneous); from 50 kg to <70 kg: 7000 IU daily (subcutaneous); from 70 kg to <100 kg: 10,000 IU daily (subcutaneous); 100 kg or above: 12,000 IU daily (subcutaneous). However, prophylactic doses were defined as follows: <100 kg: 3500 IU daily (subcutaneous); 100 kg and above: 4500 IU daily (subcutaneous).

Upon hospital admission, all patients underwent a VTE risk assessment using the PPS (≥4). The study included pathological cases meeting specific criteria. These included patients with active cancer (either with local or distant metastases or who had received chemotherapy or radiotherapy within the past six months), those confined to bed (restricted to minimal movement for physical needs for at least three days), and individuals with a history of deep vein thrombosis (excluding superficial thrombophlebitis). Additionally, patients with known thrombophilic conditions—such as deficiencies in natural inhibitors (antithrombin, protein S, protein C), Leiden factor V, the prothrombin G20210A mutation, or antiphospholipid syndrome—were included, as well as those who had undergone recent surgery or experienced trauma within the past month. Other risk factors considered were advanced age (over 70 years), heart or respiratory failure, acute myocardial infarction, ischemic stroke, hormonal therapy, obesity (BMI > 30), acute infections (e.g., respiratory, urinary tract, intra-abdominal infections, COVID-19), and rheumatological diseases. The primary endpoint was all-cause mortality during hospitalization or within 28 days after discharge. Secondary endpoints included incidence of symptomatic VTE (PE/DVT) and bleeding events (major, clinically relevant non-major, and minor) as defined by the International Society on Thrombosis and Haemostasis criteria.

We define and record the thrombotic and bleeding episodes below.

Symptomatic/Suspected VTE, including PE and DVT.

To confirm symptomatic pulmonary embolism (PE), the patient must exhibit PE symptoms along with one of the following findings: (a) A new intraluminal filling defect in subsegmental or more proximal pulmonary branches visible on a spiral CT scan or MRI scan. (b) A new intraluminal filling defect, the extension of a previous defect, or a sudden vessel occlusion seen on a pulmonary angiogram. (c) An inconclusive lung scan coupled with evidence of a new deep vein thrombosis (DVT) in the lower extremities, as documented by techniques like compression ultrasound or venography.

For confirmation of symptomatic DVT, the patient must show DVT symptoms, along with a new non-compressible venous segment detected on ultrasonography. Incidental PE is identified through one of the following criteria: (a) A new intraluminal filling defect found on a CT scan, MRI, or pulmonary angiogram. (b) An inconclusive lung scan with proof of a new DVT in the lower extremities, confirmed by compression ultrasound or venography.

Fatal PE refers to PE identified by objective diagnostic tests or autopsy, or in cases of death where the cause is not clearly determined, and DVT/PE cannot be excluded. Incidental DVT is characterized by the absence or inconclusiveness of DVT symptoms, along with a new non-compressible venous segment observed on ultrasonography.

Major, Clinically Relevant Non-Major Bleeding (CRNMB), and minor bleeding.

Major bleeding was defined as overt bleeding associated with the following: a fall in hemoglobin of 2 g/dL or more, or leading to a transfusion (of ≥2 units of packed red blood cells or whole blood), or bleeding that occurred in a critical site (i.e., retroperitoneal, intracranial, intraocular, intraspinal, intra-articular, pericardial, intramuscular with compartment syndrome or contributing to death), or bleeding contributing to death. Other clinically relevant non-major bleedings (CRNMB) were defined as noticeable bleeding that did not qualify as major bleeding but required medical intervention, resulted in unscheduled physician contact (either through a visit or phone call), led to a temporary suspension of the study treatment, or caused discomfort for the patient, such as pain or disruption of daily activities. Any other bleeding events were classified as minor.

### 2.3. Statistical Analysis

Categorical variables were summarized as counts and relative frequencies, whereas continuous variables were described using medians and interquartile ranges (IQR). Associations between categorical variables were evaluated primarily using Pearson’s chi-squared test, with Yates’ correction for continuity applied when appropriate. In instances where the dataset structure did not meet the assumptions for Pearson’s chi-squared test, Fisher’s exact test was employed as an alternative.

The normality of continuous variables was assessed using the Shapiro–Wilk and Kolmogorov–Smirnov tests. For normally distributed continuous variables, comparisons were made using Student’s *t*-test. Non-normally distributed variables were analyzed using the Mann–Whitney U test.

For multivariate analysis, a logistic regression model was utilized to evaluate the influence of independent variables. Model fit was assessed using the Hosmer–Lemeshow test, the area under the receiver operating characteristic curve (AUC), and pseudo R-squared statistics. Variables included in the logistic regression model were selected based on their clinical relevance and statistical significance in univariate analyses.

All results were reported with 95% confidence intervals (CIs). All statistical tests were two-tailed, with the level of significance set at *p* = 0.05. Data analysis and visualization were performed in RStudio 2023.06.1—Build 524 (PBC, Boston, MA, USA) using the following R packages: “dplyr” v.1.1.4, “ggplot2” v.3.5.0, “scales” v.1.3.0, “nortest” v.1.0.4, “epiDisplay” v.3.5.0.2, “broom” v.1.0.5, “lmtest” v.0.9-40, “car” v.3.1-2, “stats” v.4.3.1, “ResourceSelection” v.0.3-6, “pROC” v.1.18.5, and “pscl” v.1.5.9.

## 3. Results

### 3.1. Participant Characteristics

A total of consecutive 286 hospitalized patients were ultimately included in this study. Among them, 55.94% (160 patients) received prophylactic, while 44.06% (126 patients) received intermediate weight-adjusted doses of tinzaparin for thromboprophylaxis (Table 2). The groups did not differ significantly in their baseline and medical history characteristics, except for the history of prior arterial thrombosis, the presence of a central venous catheter, and the median Padua Score (Table 2). Among oral anticoagulants, apixaban was the most frequently prescribed agent, administered to 55.56% of patients in the first group and 33.33% in the second group, followed by rivaroxaban (22.22% and 33.33%, respectively), dabigatran (11.11% and 33.33%), and acenocoumarol (11.11% and 0%). Regarding injectable anticoagulants, enoxaparin was the most commonly used (33.33% in the first group and 50% in the second), followed by bemiparin (33.33% and 25%), tinzaparin (16.67% and 25%), and fondaparinux (16.67% and 0%).

### 3.2. Outcomes

#### 3.2.1. Thrombotic and Bleeding Events

No statistically significant differences in thrombotic or bleeding events were observed between the two study groups. No allergic or hypersensitivity reactions related to tinzaparin administration were recorded among study participants. Both thrombotic and bleeding events were more frequent in the prophylactic dose group (overall, during hospitalization, and during 28-day follow-up), although these differences did not reach statistical significance. Adverse events were minimal, with only one reported incident (Table 3).

Regarding the type of thrombotic and bleeding events, there were no statistically significant differences between the two groups (Table 3).

#### 3.2.2. Mortality and Length of Stay

**Figure 1:**
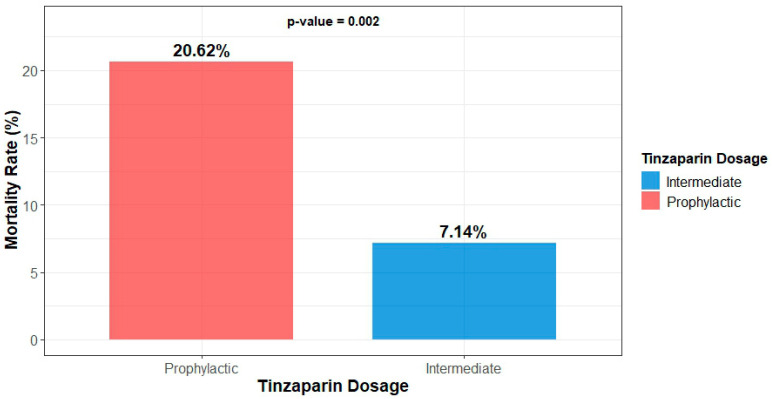
Mortality of participants by dosage group.

The mortality rate was statistically significantly higher (20.62% vs. 7.14%, *p* = 0.002) in the group receiving prophylactic doses of tinzaparin (Figure 1). However, no significant differences were noted between the two groups concerning the timing of death (during or post-hospitalization) (Table 4).

Among the 26 patients who experienced a thrombotic event, a bleeding event, or both, 5 patients died, all belonging to the prophylactic dose group. However, this difference was not statistically significant (*p* = 0.069) (Table 4).

**Figure 2:**
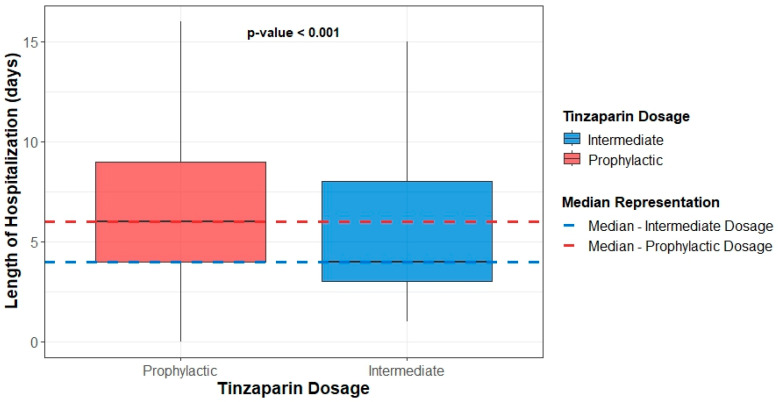
Boxplots of participants’ hospital stay duration by dosage group.

Regarding the length of hospitalization, the prophylactic dose group demonstrated a statistically significant increase in both the total hospital stay and the duration of treatment during hospitalization (Figure 2, Table 4). Finally, the ratio of hospital stay duration to treatment duration during hospitalization did not significantly differ between the two groups (Table 4). Documented infections occurred in 32 (20.0%) patients receiving prophylactic and 21 (16.7%) receiving intermediate tinzaparin (*p* = 0.42). No significant associations were observed between infection occurrence and mortality, length of stay, or treatment duration.

### 3.3. Tinzaparin Dose as an Independent Mortality Predictor

The tinzaparin dose, age, gender, length of stay (LOS), PPS, Charlson Comorbidity Index (CCI), and history of thrombotic events were analyzed for their association with mortality outcomes. Univariate analysis revealed that the type of tinzaparin dose (OR = 3.38, 95% CI: 1.61–7.78, *p* = 0.002), age (OR = 1.03, 95% CI: 1.01–1.06, *p* = 0.017), LOS (OR = 1.07, 95% CI: 1.03–1.12, *p* = 0.001), PPS (OR = 1.62, 95% CI: 1.33–2, *p* < 0.001), CCI (OR = 1.27, 95% CI: 1.13–1.44, *p* < 0.001), and history of thrombotic events (OR = 2.27, 95% CI: 1.06–4.69, *p* = 0.028) significantly influenced mortality risk. Gender was not significantly associated with mortality in the univariate analysis (OR = 0.89, 95% CI: 0.46–1.72, *p* = 0.738).

In the multivariate analysis, the type of tinzaparin dose remained statistically significant (OR = 2.58, 95% CI: 1.11–6.48, *p* = 0.035), along with age (OR = 1.04, 95% CI: 1.00–1.08, *p* = 0.033), LOS (OR = 1.10, 95% CI: 1.04–1.17, *p* < 0.001), and PPS (OR = 1.33, 95% CI: 1.02–1.75, *p* = 0.038). The CCI, gender, and history of thrombotic events were not statistically significant in the multivariate analysis. The results are presented in Table 5 for both the univariate and multivariate analyses.

## 4. Discussion

To the knowledge of the authors, this is the first study assessing the outcomes of prophylactic versus intermediate-dose tinzaparin in acutely ill medical patients with increased thrombotic risk. We found that intense prophylaxis with intermediate tinzaparin dosage showed decreased mortality compared to prophylactic dosage in our cohort. Mortality was associated with older patients treated with prophylactic doses of tinzaparin, with a longer length of hospital stay, and with a higher PPS. Incidence of thrombotic and bleeding events in patients with PPS ≥ 4 was found to be comparable between prophylactic and intermediate tinzaparin dosage.

Intense prophylaxis with intermediate tinzaparin dosage showed decreased mortality compared to prophylactic dosage in our cohort. This finding did not seem to be related to decreased thrombotic events, as respective outcome was found to be comparable between groups. It is possible, however, that this finding could be attributed to the parallel shorter LOS in these patients, possibly preventing healthcare-associated infections, including central line blood stream infections, considering that, in our cohort, patients with central venous catheter were more common in the prophylaxis group. Similarly, in this context, tinzaparin may exert its well-known pleiotropic effects, demonstrating potential immune-modulatory and antimetastatic effects beyond its anticoagulant properties, as previously shown in COVID-19 and cancer [26]. In pregnant women with cryptic recurrent miscarriages, tinzaparin therapy showed a proinflammatory effect, particularly influencing Th1/Th17-related chemokines [27], while tinzaparin’s anti-inflammatory activity has also been studied in ulcerative colitis treatment [28]. Although interesting, the clinical significance of tinzaparin pleiotropic actions in acutely ill medical patients remains unproven. The authors of this study acknowledge that no confirmed causal relationship between intermediate-dose tinzaparin and reduced infection-related complications is established for the observed mortality difference, but rather a theoretical, mechanistic explanation of the observation is proposed.

Although, in our study, increased tinzaparin dosage was related to favorable outcomes, results still remain contradictory in the global literature. Mechanistic studies describing tinzaparin’s unique anti-IIa and TFPI-releasing properties are acknowledged as exploratory and not definitive for clinical outcomes in acutely ill patients. The PROTHROMCOVID trial investigated the efficacy and safety of tinzaparin in different doses for non-critically ill COVID-19 patients. The study found no significant differences in thrombotic events, need for ventilation, or death between prophylactic, intermediate, and therapeutic doses [29]. In lung cancer patients with high thrombotic risk, an intermediate dose of tinzaparin (10,000 Anti-Xa IU) showed high efficacy and safety, with only one thrombotic event (0.7%) and ten bleeding events (7.1%) reported [30]. A retrospective study comparing enoxaparin and tinzaparin for VTE prophylaxis in acute spinal cord injury patients found that VTE was more prevalent in those receiving tinzaparin 3500 units compared to tinzaparin 4500 units or enoxaparin [31]. Overall, these studies suggest that the efficacy and safety of tinzaparin may vary depending on the patient population and dosage used.

Although our cohort of patients included patients with PPS ≥ 4, it appears that above that point increase in the score is associated with increased risk of mortality, reflected in its identification as an independent factor for poor outcomes and more prevalent in the prophylaxis dosage group. It is possible that further score increase or different cut-offs depending on the setting could affect its prognostic value. Even though PPS has been previously assessed and validated with favorable results in the prediction of high-risk patients for thrombotic risk [12,14], not all studies have confirmed its predictive utility. Prophylactic treatment in an Israeli cohort of high-risk non-surgical hospitalized patients did not significantly improve VTE incidence [32]. There was no significant difference between the study groups in terms of bleeding rates, unexplained mortality, or readmission. Additionally, PPS was not found to be an effective tool for identifying non-surgical hospitalized patients at high risk for clinically significant VTE [32]. In fact, use of the Padua score to assess VTE risk in medical wards was linked to an increased use of pharmacological prophylaxis, but it did not result in a reduction in VTE incidence or mortality rates [33].

In this setting, several other RAMS could have been used. The IMPROVE risk score has been previously utilized to assess VTE risk in 15,156 medical patients enrolled in the observational International Medical Prevention Registry on Venous Thromboembolism (IMPROVE) study [34]. In this cohort, the rate of VTE within 92 days of admission was between 0.4% and 0.5% for patients who did not have any of the four key risk factors (prior VTE, active cancer, age > 60 years, and thrombophilia). On the other hand, patients with the highest risk scores, which included from three to four of these risk factors, had VTE rates ranging from 8% to 11%, as indicated by the IMPROVE thrombosis risk model. A modified version of the IMPROVE risk score included D-dimer levels measured during the hospital stay. According to this model, a score of 2 or 3, combined with a D-dimer level more than twice the upper limit of normal, was able to identify patients at a higher risk for developing symptomatic VTE [35,36]. The GENEVA risk score was evaluated in a multi-center validation study involving 1478 hospitalized medical patients, 43% of whom did not receive thromboprophylaxis [37,38]. The 90-day risk of symptomatic VTE or VTE-related death for low-risk patients (score < 3) was 0.6% for those receiving adequate in-hospital thromboprophylaxis and 0.8% for those not receiving adequate in-hospital thromboprophylaxis. For high-risk patients (score ≥ 3), the respective risk was 3.2% for those receiving adequate in-hospital thromboprophylaxis and 3.5% for those not receiving adequate in-hospital thromboprophylaxis. A prospective study comparing these models among hospitalized patients found their predictive accuracy for VTE to be suboptimal [39]. It is thus evident that these prediction scores require further validation from independent, prospective studies before they can be used in routine clinical practice [12].

We have previously assessed the efficacy and safety of intermediate tinzaparin dosage in COVID-19 patients [40]. INTERACT was a retrospective, phase IV, observational cohort study designed to assess the clinical effectiveness and safety of a higher-than-usual prophylactic dose of tinzaparin for VTE prevention in non-critically ill COVID-19 patients with moderate disease severity. A total of 705 patients from 13 hospitals were included, with four thrombotic events (2.0%) diagnosed, consisting of three cases of pulmonary embolism (PE) and eleven cases of deep vein thrombosis (DVT). Four bleeding events (0.6%) were recorded, and 12 patients (1.7%) experienced in-hospital death. However, another randomized controlled trial showed no significant difference in efficacy between prophylactic, intermediate, and therapeutic doses of tinzaparin in non-critical COVID-19 patients [29]. Low rates of major bleeding events were also reported in this study. In brain tumor patients, a fixed prophylactic dose of tinzaparin (4500 IU daily) was found to be safe and potentially effective in reducing thromboembolic complications [41]. This study observed minimal attributable toxicity and no grade 4 or 5 CNS hemorrhages. Overall, these findings suggest that intensive dose tinzaparin may be safe for thromboprophylaxis in certain patient groups, but its efficacy may vary depending on the specific clinical context. Evidence from COVID-19 cohorts is discussed solely to illustrate tinzaparin’s broader pharmacologic actions; these findings were not extrapolated to the present non-COVID population.

Our study has a number of limitations. For example, the study was designed in a broad range of routine clinical practices in a medical ward, without specific focus on patients’ characteristics. Although CCI and PPS could uniformly summarize co-morbidities and increased risk of thromboembolic events, diversity exists among various disease; thus, unknown bias could be introduced. In our study, and in accordance with global reports, in any patient with PPS ≥ 4 attending physician could have decided on the administration of intermediate dosage. However, whether this was applied or not, was assessed on a case-by-case basis. This is reflected on the fact that even though PPS ≥ 4 was reported in all patients, median PPS significantly differed between groups as a result of random selection, adding another limitation in this context, even when applied antithrombin or anti-Xa activity was not assessed—as not regularly recommended; hence, the efficacy of intervention cannot be evaluated. Nonetheless, in the broader context, there was no selection of patients into intervention and control groups. Furthermore, all data were obtained through a retrospective review of medical records for individual patients. Evaluating the progression of acute medical conditions and their clinical outcomes is inherently complex. Various factors, such as other medications and general clinical management, are key contributors to better patient outcomes. As such, appropriate thromboprophylaxis is only one aspect of the comprehensive care required for patients with acute medical conditions. Moreover, as this was a retrospective cohort drawn from routine clinical practice, the documentation of cause of death was heterogeneous and often multifactorial, making precise attribution unreliable. Our study’s limited sample size conducted in one center precluded the inclusion of numerous factors in our multivariate analysis. As a result, the model’s findings may not accurately reflect the true relationship if a larger dataset were analyzed. Furthermore, despite the use of multivariate analysis, the influence of unmeasured confounding variables on the outcomes cannot be completely ruled out. Last, our study examined the role of tinzaparin in the prophylaxis of high-risk patients against thromboembolic events. It remains unknown whether low, intermediate or therapeutic dosages of other LMWH would behave the same way.

## 5. Conclusions

In conclusion, intermediate tinzaparin dosage showed favorable outcomes compared to prophylactic dosing schemes in acute medically ill high-risk patients for VTE. While intermediate dose tinzaparin demonstrated lower mortality, VTE and bleeding rates remained comparable between dosing groups, highlighting the need for randomized studies to confirm these findings. Our results underline the importance of the tailor-made management of high-risk patients, in whom we commonly universally administer prophylactic dosages. In this population, management on a case-by-case basis seems more appropriate, while the accurate and timely identification of patients that will benefit intensified doses is pivotal to ensure best the outcomes for our patients. This single-center retrospective study suggests that intermediate-dose tinzaparin may be associated with lower mortality among high-risk medical inpatients. Given its observational design and inherent limitations, the findings should be interpreted as hypothesis-generating and warrant confirmation in prospective multi-center trials.

## Figures and Tables

**Table 1:** Padua VTE RAM in medical inpatients.

Padua VTE RAM: Score ≥ 4 Indicates High VTE Risk	Points
Active cancer	3
Reduced mobility	3
Known thrombophilic condition	3
Previous VTE (excluding superficial thrombophlebitis)	3
Recent Trauma and/or Surgery (<1 month)	2
Elderly age (>70 years/old)	1
Heart and/or Respiratory Failure	1
Ongoing hormonal treatment	1
Acute myocardial Infarction or Ischemic Stroke	1
Obesity (Body Mass Index > 30)	1
Acute infection and/or rheumatologic disorder	1
VTE: Venous thromboembolism; RAM: Risk Assessment Model;	

**Table 2:** Participant characteristics and medical history.

Characteristic		Tinzaparin Dose		
		Prophylactic (*n* = 160)	Intermediate (*n* = 126)	*p*
**BASELINE CHARACTERISTICS**				
**Age (years)**		75 (64–82.25)	76 (66–83.75)	0.654
**Gender (Male)**		88 (55%)	55 (43.65%)	0.074
**Postmenopausal women (Yes)**		(*n* = 72)	(*n* = 71)	
		66 (91.66%)	64 (90.14%)	0.546
**Body Mass Index**		24.69 (23.38–27.61)	24.49 (22.86–26.30)	0.157
**Charlson Comorbidity Index**		6 (4–9)	6 (4–7)	0.088
**THROMBOTIC/BLEEDING HISTORY**				
**Number of Thrombotic Episodes**		(*n* = 160)	(*n* = 126)	
	None	135 (84.38%)	98 (77.78%)	0.203
	One	23 (14.37%)	26 (20.63%)	0.216
	Two	2 (1.25%)	2 (1.59%)	1
**Type of Thrombotic Episodes**				
	Stroke	12 (7.55%)	11 (8.73%)	0.884
	Myocardial infarction	9 (5.66%)	9 (7.14%)	0.790
	Pulmonary embolism	1 (0.63%)	2 (1.59)	0.584
	Superficial vein thrombosis	1 (0.63%)	0 (0%)	1
	Deep vein thrombosis	1 (0.63%)	1 (0.79%)	1
	Splanchnic vein thrombosis	0 (0%)	1 (0.79%)	0.440
	Other arterial thrombosis	0 (0%)	6 (4.76%)	0.006
**Bleeding Episode**				
	Yes	7 (4.4%)	7 (5.56%)	0.863
**Type of Bleeding Episodes**				
	Overt bleeding requiring medical intervention	3 (1.89%)	3 (2.38%)	1
	Clinically evident with a hemoglobin decrease ≥2 g/dL within 24 h	1 (0.63%)	0 (0%)	1
	Clinically evident with transfusion of ≥2 blood units	1 (0.63%)	0 (0%)	1
	Clinically evident in a critical location (e.g., intracranial, retroperitoneal)	1 (0.63%)	1 (0.79%)	1
	Causing unplanned medical contact	1 (0.63%)	2 (1.59%)	0.585
	Causing discomfort or limiting daily activities	0 (0%)	1 (0.79%)	0.422
**HISTORY OF LONG-TERM ANTICOAGULANT USE**				
**Oral Anticoagulant Use**		9 (5.62%)	6 (4.76%)	0.953
**Injectable anticoagulants**		6 (3.75%)	8 (6.35%)	0.462
**Aspirin or Other Antiplatelet Use**		40 (25%)	36 (28.57%)	0.586
**RISK FACTORS FOR NEW THROMBOSIS**				
**Padua Prediction Score**		6 (5–7)	5 (4–6)	<0.001
**Smoking (Yes)**		67 (41.88%)	42 (33.3%)	0.175
**Limited mobility (Yes)**		123 (76.88%)	104 (82.54%)	0.303
**Phlebitis/Varicose veins (Yes)**		1 (0.63%)	1 (0.79%)	1
**Central venous catheter**		20 (12.5%)	3 (2.38%)	0.003
**Antiphospholipid antibodies**		2 (1.27%)	0 (0%)	0.504
**Recent surgery (Yes)**		7 (4.37%)	4 (3.17%)	0.760

**Table 3:** Thrombotic and bleeding events among participants during the study.

Characteristic		Tinzaparin Dose		
		Prophylactic (*n* = 160)	Intermediate (*n* = 126)	*p*
**TOTAL THROMBOTIC/BLEEDING EVENTS**				
**Thrombotic**		7 (4.37%)	3 (2.38%)	0.520
	Deep vein thrombosis	4 (57.14%)	0 (0%)	0.200
	Splanchnic thrombosis	2 (25.57%)	1 (33.33%)	1
	Pulmonary embolism	1 (14.29%)	2 (66.67%)	0.183
**Bleeding**		13 (8.13%)	4 (3.17)	0.132
	Minor bleeding	6 (46.15%)	2 (50%)	1
	Major bleeding	7 (53.85%)	2 (50%)	1
**THROMBOTIC/BLEEDING EVENTS DURING HOSPITALIZATION**				
**Thrombotic**		5 (3.12%)	1 (0.79%)	0.233
**Bleeding**		9 (5.62%)	2 (1.59%)	0.119
**THROMBOTIC/BLEEDING EVENTS DURING 28 DAY FOLLOW-UP**				
		(*n* = 134)	(*n* = 119)	
**Thrombotic**		2 (1.49%)	2 (1.71%)	1
**Bleeding**		4 (2.99%)	2 (1.69%)	0.687

**Table 4:** Mortality, hospital stay, and treatment data.

Characteristic		Tinzaparin Dose		
		Prophylactic (*n* = 160)	Intermediate (*n* = 126)	*p*
**MORTALITY**				
**Mortality (Yes)**		33 (20.62%)	9 (7.14%)	0.002
	During hospitalization (% among overall mortality)	26 (78.79%)	7 (77.78%)	1
	Post-hospitalization (28 day follow up) (% among overall mortality)	7 (21.21%)	2 (22.22%)	1
**MORTALITY WITH EPISODE PRESENCE (THROMBOTIC/BLEEDING)**				
**Thrombotic event–Mortality**		0 (0%)	0	
**Bleeding event—Mortality**		4 (12.12%)	0	
**Thrombotic and Bleeding event—Mortality**		1 (3.03%)	0	
**Total episodes—Mortality**		5 (15.15%)	0	0.069
**DURATION OF HOSPITALIZATION/TREATMENT**				
**Length of Stay (days)**		6 (4–9)	4 (3–8)	<0.001
**Length of treatment during hospitalization (days)**		5 (3–8.25)	3 (2–7)	0.003

**Table 5:** Univariate and multivariate analysis of factors correlated with mortality.

	Unadjusted Analysis			Adjusted Analysis		
	Odds Ratio	95% CI	*p*-Value	Odds Ratio	95% CI	*p*-Value
Tinzaparin Dose (Prophylactic)	3.38	1.61–7.78	0.002	2.58	1.11–6.48	0.035
Age	1.03	1.01–1.06	0.017	1.04	1.00–1.08	0.033
Gender (Male)	0.89	0.46–1.72	0.738	0.89	0.42–1.87	0.762
LOS (Length of Stay)	1.07	1.03–1.12	0.001	1.10	1.04–1.17	<0.001
Padua Prediction Score	1.62	1.33–2.00	<0.001	1.33	1.02–1.75	0.038
Charlson Comorbidity Index (CCI)	1.27	1.13–1.44	<0.001	1.13	0.96–1.33	0.135
History of Thrombotic Event (Yes)	2.27	1.06–4.69	0.028	1.79	0.74–4.20	0.183

Reference categories: Tinzaparin Dose = Intermediate, Gender = Female, History of Thrombotic Event = No.

## Data Availability

The data presented in this study are available on request from the corresponding author due to privacy and ethical restrictions.

## Abbreviations

The following abbreviations are used in this manuscript:

CCI	Charlson Comorbidity Index
CNS	Central Nervous System
COVID-19	Coronavirus Disease 2019
DVT	Deep Vein Thrombosis
LMWHs	Low Molecular Weight Heparins
LOS	Length of Stay
OR	Odds Ratio
PE	Pulmonary Embolism
PPS	Padua Prediction Score
RAMs	Risk Assessment Models
VTE	Venous Thromboembolism
CI	Confidence Interval
IU	International Units
RCT	Randomized Controlled Trial
CRNMB	Clinically relevant non-major bleedings
TFPI	Tissue Factor Pathway Inhibitor
SC	Subcutaneously
OD	Once Daily
IQR	Interquartile range
BMI	Body Mass Index
IMPROVE	International Medical Prevention Registry on Venous Thromboembolism
